# ALICE: An open-source tool for automatic measurement of phoneme, syllable, and word counts from child-centered daylong recordings

**DOI:** 10.3758/s13428-020-01460-x

**Published:** 2020-09-01

**Authors:** Okko Räsänen, Shreyas Seshadri, Marvin Lavechin, Alejandrina Cristia, Marisa Casillas

**Affiliations:** 1 Unit of Computing Sciences, Tampere University, P.O. Box 553, FI-33101 Tampere, Finland; 2 Department of Signal Processing and Acoustics, Aalto University, Espoo, Finland; 3 Laboratoire de Sciences Cognitives et de Psycholinguistique, Département d’études cognitives, ENS, EHESS, CNRS, PSL University, Paris, France; 4 INRIA, Paris, France; 5 Max Planck Institute for Psycholinguistics, Nijmegen, Netherlands

**Keywords:** Child-centered audio, Word count estimation, Language development, Speech processing, Speaker diarization, LENA

## Abstract

Recordings captured by wearable microphones are a standard method for investigating young children’s language environments. A key measure to quantify from such data is the amount of speech present in children’s home environments. To this end, the LENA recorder and software—a popular system for measuring linguistic input—estimates the number of adult words that children may hear over the course of a recording. However, word count estimation is challenging to do in a language- independent manner; the relationship between observable acoustic patterns and language-specific lexical entities is far from uniform across human languages. In this paper, we ask whether some alternative linguistic units, namely phone(me)s or syllables, could be measured instead of, or in parallel with, words in order to achieve improved cross-linguistic applicability and comparability of an automated system for measuring child language input. We discuss the advantages and disadvantages of measuring different units from theoretical and technical points of view. We also investigate the practical applicability of measuring such units using a novel system called Automatic LInguistic unit Count Estimator (ALICE) together with audio from seven child-centered daylong audio corpora from diverse cultural and linguistic environments. We show that language-independent measurement of phoneme counts is somewhat more accurate than syllables or words, but all three are highly correlated with human annotations on the same data. We share an open-source implementation of ALICE for use by the language research community, enabling automatic phoneme, syllable, and word count estimation from child-centered audio recordings.

## Introduction

The use of (day)long child-centered audio recordings from children’s natural environments is becoming one of the standard methods for studying child language acquisition of spoken languages. By using a wearable recorder to capture what children hear in their daily lives, researchers can characterize the quality and quantity of language input and infant–caregiver interaction that children experience, and analyze how such factors may relate to later developmental outcomes (e.g., Gilkerson et al., 2018; Romeo et al., 2018; Suskind et al., 2016; Caskey et al., 2014; Ramírez-Esparza, García-Sierra, & Kuhl, 2014; Weisleder & Fernald, 2013). However, the amount of audio data collected with wearable recorders from a population of learners easily surpasses the capacity of any single research lab to comprehensively manually annotate the data for all variables of interest. This means that automatic or semiautomatic tools are essential for processing and analyzing such recordings (see Casillas & Cristia, 2019, for a review).

One key measure of interest is the amount of linguistic input that a child hears within a given time period (e.g., specific time of the day, within the full day, or in a specific environment such as daycare or at home). The existing standard solution to collecting and analyzing child-centered recordings is the widely adopted LENA system (Xu et al., 2008; Gilkerson & Richards, 2009). It consists of a physical recording device and associated software for automatically analyzing a number of variables from the data, including estimation of the number of words spoken by adults in the vicinity of the child, in addition to detecting child vocalizations and conversational turns. However, LENA is proprietary and expensive, and its core technology is aging with respect to the cutting edge in automated speech processing. In addition, LENA is optimized for American English, which means that its absolute word count estimates tend to be more accurate for English than for other languages (though relative counts in within-corpus comparisons are still usually reliable; see Räsänen et al., 2019, for a summary). In order to address these shortcomings of LENA, an open-source alternative for automatic word count estimation was proposed by Räsänen et al. (2019) (hereafter referred to as “*WCE-R*”). Instead of using a single static model for word count estimation, WCE-R can be adapted to any target language of interest by using a few hours of orthographically transcribed child-centered audio. As a result, WCE-R outperformed LENA on three out of four tested dialects of English, and achieved similar performance on multiple languages other than English (see Räsänen et al., 2019, for details). However, while the WCE-R adaptation procedure improves the cross-linguistic performance of the system, the requirement of transcribed domain-specific data also greatly limits the practical usability of WCE-R as a standardized tool for developmental research, not least since its performance ultimately depends on the quality and representativeness of the adaptation data used for the domain of interest.

A fundamental challenge with both WCE-R and LENA is that accurate word count estimation in any given language necessarily requires at least some knowledge of the lexicon and phonology of that language. Integrating such knowledge into the system for all of the world’s approximately 7000 languages (number from Coupé et al., 2019) is simply not feasible. This is especially true for low- resource languages, for which orthographically transcribed data may be sparse or may not exist at all. LENA’s only way to cope with this issue is to simply assume that all speech is American English, whereas WCE-R uses the data-driven adaptation discussed above. Arguably, neither of the approaches is ideal from the cross-corpus (cross-linguistic) analysis point of view, where sensitivity and accuracy of the unit count estimates would ideally be similar across different datasets.

An independent, but equally important issue is whether words are the linguistic units that we should be counting in the first place. While a great deal of current developmental theory has built up around children’s vocabulary development, including the implications of vocabulary growth for syntactic development (e.g., Frank et al., in prep; Brinchmann, Braeken, & Lyster, 2019; Marchman, Martínez-Sussmann, & Dale 2004; Bates & Goodman, 1997; Lieven, Pine, & Baldwin, 1997), the predictive value of input word count for long-term language development is still unclear, and has been primarily limited to studies of English (e.g., Gilkerson et al., 2018; Caskey et al., 2014; Weisleder & Fernald, 2013; Hurtado, Marchman, & Fernald, 2008; Hoff & Naigles, 2002; Hart & Risley, 1995). Considering, too, the basic fact that human languages vary in how words are composed (e.g., carrying one or multiple morphemes), it is apparent that word count is far from optimal when trying to compare input rates cross-linguistically. One could instead measure other linguistic units such as *phones* (or *phonemes*), *syllables* (or *morphemes*), *utterances*, or even simply the *total duration* of speech (cf., DeAnda et al., 2016). However, we know even less about how these units relate to child language development, so it is not obvious whether they adequately serve as meaningful substitutes for words.

Coming back to technical concerns of automated detection, it is also unclear whether these alternative units can be measured robustly despite the noisy soundscapes of typical daylong audio recordings, and with similar accuracy across languages and cultural settings (e.g., (sub)urban post-industrial vs. rural subsistence farming). Notably, within a single linguistic population, phoneme, syllable, and word counts are likely to be highly correlated on the time scales typically used in linguistic input analyses (e.g., multiple hours), thereby allowing cumulative counts of one type of unit to be obtained from another through simple linear scaling. Our point for present purposes, however, is that it is not at all obvious whether one generic linguistic unit estimator can perform equally well *across* languages, or whether the estimator’s output could be directly compared *between* languages.

In order to tackle these questions, the present paper considers whether more cross- linguistically reliable measures of spoken child language exposure could be established by using linguistic units other than words. More specifically, we investigate how much the use of phonemes, syllables, or words as the basic units of language input makes sense from linguistic, developmental, and technological viewpoints. We focus especially on the cross-linguistic comparability and compatibility of the measures as well as the accuracy at which phoneme, syllable, and word counts can be derived from child-centered daylong audio recordings when using language-dependent and language-independent approaches. As a part of this work, we present our open-source system, Automatic LInguistic unit Count Estimation (ALICE^1^), and use it to investigate the feasibility of estimation for the different units on seven different child-centered audio corpora from various language environments.

We start with theoretical and technical considerations for measuring syllables versus words (the next section. Following the earlier work on LENA (Xu et al., 2008) and WCE-R (Räsänen et al., 2019), we then describe our system for estimating phoneme, syllable, and word counts from acoustic speech (Methods). Performance of the system is then evaluated in within- and across-language linguistic unit count estimation tasks (Experiments), including a comparison and combination of different intermediate signal representations in the estimation tasks. Finally, we discuss the implications of our results.

## Measuring phone(me)s, syllables, and words

The overall aim of this paper is to investigate which linguistic units one should be measuring for spoken input, when the ultimate goal of research is to characterize both (a) the language input that children hear in their daily environments, and (b) how that input relates to other variables of interest in the child’s development. An ideal solution has all the following desiderata: 1) the quantity of the chosen units is highly relevant to the learning mechanisms linking linguistic input to language development, 2) the units have a meaningful and interpretable relationship to linguistic theory, 3) the units of interest can be measured automatically from child-centered daylong audio with comparable accuracy across languages, and 4) the automatic measurement system operates out-of-the-box on any given dataset. Additional desired characteristics include 5) reasonable computational requirements so that an actual implementation of the system can be operated in typical computing environments, and 6) public availability of the required components to openly disseminate the system for researchers in the field, improving reproducibility as well as comparisons across studies. Finally, there has to be 7) a gold-standard reference available for the units of interest on relevant data in order to benchmark and validate an automatic system for their estimation.

Starting from these considerations, we chose three candidate units of interest: phonemes, syllables, and words. Since our primary interest is in speech from language-proficient adult caregivers, the remainder of the paper will use the term “adult linguistic unit counts” (ALUC) to refer to any of these three unit types interchangeably. This section first discusses the technical factors in measuring such units from real-world audio data, and then brings up a number of theoretical considerations with respect to their analysis and use in quantifying child language input.

### Technical considerations

One of the main challenges in developing a unit count estimator for daylong audio recordings is the complexity and noisiness of the acoustic data on which the analyses are conducted. The microphone worn by the child captures all types of sounds in their vicinity, including various types of stationary and non-stationary background noises, overlapping speech, and far-field and reverberant speech that can be difficult to comprehend even for a human listener. For instance, Räsänen et al. (2019) reported an average speech signal-to-noise ratio (SNR) of 0 dB across the same set of naturalistic daylong corpora used in the present study (see Methods), which is substantially lower than the noise conditions for which speech technology algorithms are typically optimized (e.g. SNR of ~10–40 dB). There is also substantial variability in many other properties of the recordings, including signal conditions and the amount of speech versus non-speech audio, both between individual children and across different corpora. The second main challenge is that the speech within the recordings and across corpora can contain different languages, whereas tools related to linguistic unit detection, such as automatic speech recognition (ASR), are normally tailored for one language at a time.

Another central characteristic of the automatic linguistic unit count estimation process is that the variable of interest is a cumulative sum of individual tokens across a period of time (e.g., total number of words heard at daycare vs. home). This means that accurate identification of individual tokens (e.g., lexemes) is not necessary. Instead, the problem can be solved by finding a set of audio signal descriptors that accumulate approximately linearly as a function of the number of units of interest, checking that this property is satisfied at the time scales of interest. Once such correlates are found, they can be mapped to an expected value of the desired unit count using a simple linear model, a strategy utilized in all existing work on ALUC, including LENA (Xu et al., 2008; Ziaei et al., 2016; Räsänen et al., 2019). For instance, LENA does not recognize individual spoken words, but simply calculates the number of vowels, consonants, and silences in the input, as detected by an automatic phone recognizer trained for American English. These counts are then mapped to the most likely corresponding word count using a linear model, also tuned for American English. The WCE-R system by Räsänen et al. (2019) performs a similar process but using syllables instead of phones as the basic unit of measurement, and using a linear model trained separately for each language of interest. In the same way, features such as total duration of speech could be mapped onto the expected number of linguistic units simply by considering the average speaking rate of the given units in that language. Even though this would ignore speaker-dependent differences in the speech rate, the estimate would still be very highly correlated with the true unit counts, as any short-term variations in the speaking rate would tend to average out across a measurement period of anything from several minutes to several hours (see also Räsänen et al., 2019).

What the above means from a technical point of view is that the unit of measurement would ideally be directly recognizable from the acoustic signal in a language-independent manner. Alternatively, the unit should be strongly linearly correlated with measurable characteristics of acoustic speech, and this correlational relationship should not change across languages, dialects, speakers, or speaking conditions. Additionally, the acoustic measurement process should be robust against noise overlapping with speech, but also against any non-overlapping non-speech sounds, as it is not currently technically feasible to carry out perfectly accurate speech segmentation and speaker identification from naturalistic audio recordings (e.g., Sun et al., 2018; Sell et al., 2018; Ryant et al., 2019; Sahidullah et al., 2019; Cristia et al., 2020).

From this perspective, the main disadvantages of word counting are that direct words cannot feasibly be estimated across all languages and that indirect word count estimates are not comparable across languages. First, direct measurement of word counts requires a full automatic speech recognition (ASR) system, which is not necessarily feasible or accurate for low-resource or even unwritten languages (i.e., the vast majority of the world’s languages). Even if we did have sufficient annotated data for each of the world’s languages, integrating all these language-specific ASR systems into a common one such that performance and output were comparable across languages would be an immense technical challenge. Note that these same restrictions apply to morphological analysis, which would otherwise be a much better approach for cross-linguistic comparability in number of expressed units of meaning (see Allen & Dench, 2015, for a discussion). The only feasible choice, then, is to indirectly estimate word (or morpheme) counts on the basis of other acoustic cues. As far as we know, the relationship between measurable correlates of words (e.g., automatically detected phone(me)s or syllables) and the corresponding word counts themselves is relatively systematic within language, especially at larger time scales, as demonstrated by LENA and WCE-R. However, the exact relationship is also necessarily dependent on the language in question, as there are no universal acoustic or perceptual cues for words or word boundaries. For instance, LENA AWCs are optimized for American English, but the *absolute* word count accuracy varies for other languages. On the other hand, LENA-based *relative* AWCs *within* a corpus are still quite systematic, and are therefore useful for analyses *within* the given corpus (see Räsänen et al., 2019, for a summary)

In contrast to words, syllables are potentially easier to detect across languages. This is because the basic syllabic structure—temporal alternation of low- and high-sonority speech sounds—is generally present in all spoken languages as the physical consequence of articulatory sequencing. Since the fluctuation of sonority can be operationalized in terms of, e.g., speech amplitude or loudness modulations, automatic syllabification of speech can be performed in a language-independent manner (e.g., Mermelstein, 1975), with a number of algorithms reaching relatively comparable accuracy across different languages (Räsänen, Doyle, & Frank, 2018a; Seshadri & Räsänen, 2019). However, one problem is that the amplitude modulations of speech can be easily confused with similar modulations in other sounds, making the sonority-based algorithms potentially susceptible to noisy audio. Nevertheless, this downside of “traditional” syllabification algorithms can potentially be tackled with machine learning approaches. By training the system using a large number of syllables from different languages (potentially from different noise conditions), a machine learning algorithm may learn a general solution for syllable counting that tolerates non-speech audio content better than the traditional signal processing approaches (e.g., Räsänen, Seshadri, & Casillas, 2018b; Seshadri & Räsänen, 2019). This makes syllables a potential candidate unit that could be estimated directly from speech instead of using correlated features such as speech duration or phone counts as linear correlates of syllable counts. Still, due to the granularity of syllables as basic units of detection, individual missing or inserted syllables can lead to large relative errors in the estimated counts of short utterances. However, if the syllabification errors are unbiased (i.e., the insertions and deletions are equally frequent) and infrequent enough, the errors may average out harmlessly at larger analyzed time scales (e.g., multiple hours).

Finally, phone (or phoneme) counting has certain theoretical technical advantages over words. Even though different spoken languages have different phoneme and phone inventories, they can be seen as subsets of a larger set of possible spoken segments used in human language (Ladefoged & Maddieson, 1996). In addition, if the primary goal is to count the units, not to identify them precisely, an automatic phone recognizer trained on any language should be reasonably good at identifying the vowel and consonant segments even if the exact narrow phonetic category is not correctly recognized (cf. LENA phone recognition front-end). When training the recognizer on more languages, the system should also become more sensitive to vowel-to-vowel and consonant-to-consonant transitions, even if the input corresponds to a language with mismatching phonology to any of the training languages. This could then enable language-independent phone(me) counting. Since phones are smaller units than syllables, individual insertions or deletions in the detection process are not as harmful to the overall estimate as at the syllabic level. If the errors are unbiased, they also tend to average out over time similarly to syllable detection.

To summarize, there are certain technical factors that could make language-independent measurement of phone(me)s and syllables easier than measurement of words, at least in theory. In contrast, measurement of linguistic units such as morphemes or input characteristics such as syntactic complexity is more difficult to automate in a language-independent manner due to their high dependency on the linguistic characteristics of the language in question. However, the practical feasibility of phone(me)s and syllables also greatly depends on the quality of the available speech technology solutions to identify such units from child-centered recordings. This potential limitation is amplified by the fact that the existing training data for automatic phone or syllable recognition systems comes mostly from audio corpora with very different acoustic and speaker characteristics from those of child-centered audio recordings. In addition, the question is not only of *what can be measured*, but *what should be measured* when child language development is of interest.

### Linguistic and developmental considerations

Accurate estimates of how much language children encounter are critical to outlining the quantity and types of linguistic evidence needed to support language learning. On the level of daylong recordings, one can theoretically combine average input quantity measures with independently estimated average rates for a given linguistic phenomenon (e.g., syntactic word types) to infer how much exposure children typically have to that phenomenon (but see Bergelson et al., 2019, for warnings). On the level of individual utterances, unit estimates serve as an index of utterance complexity, which itself has been suggested as a predictive measure of later language outcomes (e.g., Vasilyeva, Waterfall, & Huttenlocher, 2008; Huttenlocher et al., 2007). As these two examples demonstrate, quantity measures can, in principle, be linked to contentful, quality-like features of children’s language environments while also preserving comparable quantitative estimates across children.

It is not yet known what linguistic units are most relevant for measuring the quantity of children’s language input during development, either across human languages or within a single language (i.e., given a language’s particular structural properties). Current standard practice is to estimate children’s linguistic input in words (e.g., 7000 words per day/440 words per hour). This practice gained popularity after Hart and Risley’s landmark (1995) study of children’s at-home speech environments and was later amplified by its inclusion in the LENA system’s automated analysis (Xu et al., 2008; Gilkerson & Richards, 2009). The current dominance of word counts in measuring linguistic input is then arguably due, in large part, to convenience.

As mentioned above, an ideal ALUC measure should perform well in at least two critical dimensions: it should closely relate to later language outcomes, and it should be as comparable as possible across languages. Note, however, that in choosing a linguistic unit for analysis, these two features may be weighed against each other differently. As we will see, the research question, type of data being used, and populations under study are all relevant considerations in focusing on a unit type. That aside, given these two core desiderata, we briefly consider the advantages and disadvantages of four measures of input quantity: words, syllables, segments, and duration. These four measures are, by nature, highly correlated with each other within languages but, as we will see, differ in their cross-linguistic and developmental applicability.

Words are meaning-bearing units, and are therefore tempting as a measure of the quantity of meanings that the child has encountered. Vocabulary knowledge and speed of lexical retrieval are some of the most commonly used indices of overall language development in observational, experimental, and questionnaire-based studies, and linguistic input count in words makes a convenient parallel for considering the acquisition of these phenomena. Word counts have also been linked to later language development in English and Spanish (Gilkerson et al., 2018; Caskey et al., 2014; Weisleder & Fernald, 2013; Hurtado, Marchman, & Fernald, 2008; Hoff & Naigles, 2002; Hart & Risley, 1995), fulfilling the first of our two desiderata above. A major issue with word counts, however, is that they are not meaningful for broad cross-linguistic comparisons. For instance, synthetic languages such as Finnish tend to have words composed of multiple bound morphemes, while analytic languages like English typically communicate the same meaning using multiple words (e.g., “*Menisimmekö*?”, “*Shall we go*?"”, or “*Käsittääksemme*”, “*In order for us to understand*”/“*According to our understanding*”). Finally, early on in development, infants are unlikely to be perceiving the speech stream in “words,” at least in its entirety (Bergelson & Aslin, 2017; Bergelson & Swingley 2012); therefore, a different measure of input may be appropriate at these initial stages of linguistic knowledge.

Syllables are an attractive alternative to words in measuring linguistic input. Syllables are less theoretically loaded than words, and may therefore more easily apply cross-linguistically. Consider again the case of Finnish and English (“*Menisimmeköhän*?” vs. “*I wonder if we shall go*”); the English realization of the utterance has five more words, but only one more syllable than the Finnish one (see also Allen & Dench, 2015, for consideration of these issues with respect to child-produced speech). The comparative advantages of syllables over words are similarly obvious for multilingual speech environments. Syllables are also the minimal unit on which prosodic structure is carried, meaning they carry critical weight in the recognition of lexical and syntactic boundaries. And while young infants do not yet know many words, they could use syllables to segment words and infer syntactic structures from the speech stream. Experimental evidence also suggests that syllables are available as a perceptual unit to newborns, possibly even more so than phonological segments (e.g., Jusczyk & Derrah, 1987; Bertoncini et al., 1988; Bijeljac-Babic et al., 1993; Swingley, 2005; see also Räsänen, Doyle, & Frank, 2018a). Perhaps for this reason, many current computational models assume that infants use syllables as the basis for speech segmentation and non-adjacent dependency learning (e.g., Poletiek et al., 2018; French, Addyman, & Mareschal, 2011; Swingley, 2005; Perruchet & Vinter, 1998). Indeed, infants and adults alike have been shown to segment strings and learn structures in a number of experimental studies, all theoretically based on computations over syllables (Saffran et al., 1996, 1997; Pelucchi et al., 2009; Black & Bergmann, 2017). That said, infants’ ability to learn non-adjacent dependencies is difficult to accomplish on the basis of syllable information alone, and benefits from the addition of phonological cues (Newport & Aslin, 2004), suggesting that their predictive weight as a perceptual unit for learning may be more limited than initially assumed. Syllables are also more subject to deletion than are whole words in fluent speech, though they are more resistant to deletion than individual phonological segments (Greenberg, 1999). Regardless, variation in the likelihood of syllable deletion across languages may cause trouble for the applicability of syllables as a central measure of linguistic input.

Phonological segments are even smaller units available to infants, though perhaps their availability as distinct perceptual units comes later than it does for syllables (see Swingley, 2005, for a review). Segments are minimally theoretically loaded and so could be argued to be the most fair linguistic estimate type across diverse recording contexts and languages. As mentioned above, distributional phonological cues have also been shown to be useful in computational modeling (Monaghan & Christiansen, 2010), corpus analysis (Hockema, 2006), and experiments (Newport & Aslin, 2004) to aid infants’ ability to extract strings and structures from running speech. In speech segmentation, phonological cues derived from segment-level distributions such as vowel and consonant harmony are used by infants to extract strings (e.g., Kabak et al., 2010; Mintz et al., 2018). For a number of languages, including English, Turkish, Mandarin, Dutch, French, and Japanese, phonological features and syntactic classes correspond such that, even at the segmental level, infants may already get a limited view into syntactic structure (Sereno and Jongman, 1995; Shi et al., 1998; Monaghan, Christiansen, & Chater, 2007). However, of all the linguistic units discussed so far, segments are the most likely to be deleted in reduced speech (Greenberg, 1999). The effects of reduction on segments compared to syllables or words may also vary cross-linguistically. Developmentally, it is unclear whether children come to the world perceiving segments as privileged units (as opposed to, e.g., syllables; see above). At the same time, segments are quite far removed from communicative meanings, and their standalone explanatory power for later language outcomes is yet to be determined.

Speech duration is the least theoretically loaded approach to quantifying the input and therefore optimally serves the goal of minimizing bias across languages, but also comes at the cost of having very little explanatory depth and, perhaps also, minimal predictive power when it comes to later language outcomes. That said, speech duration can be more simply and reliably measured from the speech signal compared to the other units discussed so far. Duration can also yield surprising insight if prior estimates exist regarding information transfer. For example, Coupé et al. (2019) found that the average information rate of speech recorded from read-aloud texts was approximately the same (~39 bits/s) across 17 different languages. This suggests that duration of speech could, in fact, reliably index how much information (“meaning”) is being transferred. That said, it is worth bearing in mind that the results were based on read-aloud texts and not on spontaneous child-directed speech and that the broad conclusions hide significant between-speaker variability. Even if these results held up robustly for child-directed speech, we might still encounter the problem that children’s ability to perceive and compute different linguistic units may change as they get older; in other words, the interpretable bitrate might also change with development.

In sum, an ideal ALUC measure for language development should relate to later language outcomes and should be usable in comparative cross-linguistic or multilingual input work. The four options we considered here vary in their utility given what assumptions we can make about the child’s developmental state, their validity for direct comparison across linguistic contexts, their likely value in illuminating the underlying mechanisms of language learning, and their correspondence to existing tools and models independently designed to assess the process and outcomes of language learning. Therefore, when we consider the development of an ALUC estimator, the best approach may be one that provides an array of options, rather than focusing on a single one (e.g., adult word counts).

## Methods

**Fig. 1:**
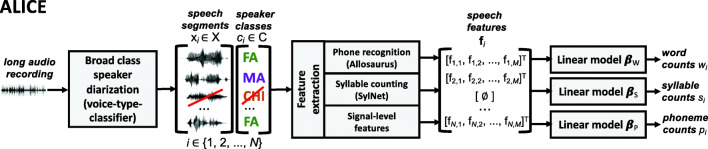
Block schematic of ALICE for automatic linguistic unit count estimation. The current example demonstrates a use case where only adult speech is measured, i.e., speech detected from children (CHI) is ignored in the process

Our system for ALUC estimation is called Automatic LInguistic unit Count Estimator (ALICE). The basic architecture of ALICE is shown in Fig. 1. ALICE is conceptually based on the earlier systems in Räsänen et al. (2019), Ziaei et al. (2016), and the LENA system (Xu et al., 2008), fusing many of their ideas into a common publicly available open-source pipeline. ALICE also extends the child- centered diarization pipeline called DiViMe described in Le Franc et al. (2018), but operates in a generic Python environment instead of a Linux virtual machine. ALICE also uses a more powerful speech detection and speaker diarization front-end than the earlier DiViMe-system.

The ALICE pipeline consists of three main components: 1) a module responsible for speech detection and speaker diarization, 2) feature extraction from detected speech segments from talkers of interest, and 3) mapping of features into linguistic units of interest using a set of linear models, one model for each unit type. The main processing strategy undertaken in ALICE is to utilize language- independent feature extraction modules for ALUC estimation. For the purpose of the present experiments, ALICE will be presented with several parallel feature extraction modules in order to compare their usefulness in the ALUC estimation tasks. In the open-source distribution of ALICE, only the best compromise between ALUC estimation performance and computational speed is provided, as defined in the final discussion. Different system modules are described in more detail in the following subsections.

In our earlier work, we also investigated the use of speech enhancement as a preprocessing step to alleviate the impact of unwanted noise in child-centered recordings (Mital, 2019; Mital & Räsänen, submitted). However, the performance gains from enhancement were found to be very minor when combined with detectors and classifiers that can be directly trained to operate on the noisy child-centered data. Therefore, no dedicated speech enhancement module is included in ALICE.

### Broad class diarization

The first stage of ALICE consists of a broad class diarization module. The goal of the module is to extract speech segments and the corresponding talker type information from an input audio waveform that contains all sounds in the surroundings of the child wearing the microphone. In the present context, broad class diarization refers to classification of speech segments into one of four talker categories: 1) female adult (“FA”), 2) male adult (“MA”), 3) key child (“KCHI”), and 4) other children (“CHI”). FA and MA speakers together form the main category of interest for estimation of adult speech heard by a child. KCHI includes speech by the child wearing the microphone, and CHI covers other children. After diarization, all non-speech segments and speech from undesired talkers (CHI and KCHI when measuring adult input) are discarded.

The diarization system that ALICE uses is called *voice type classifier* (Lavechin et al., (in press) submitted; Lavechin, 2020), which originates from large-scale collaborative development efforts carried out at the JSALT 2019 workshop (Garcia et al., 2020). Technically speaking, it is based on a neural network architecture called SincNet (Ravanelli & Bengio, 2018), and the model is trained on approximately 200 hours of daylong child-centered audio recordings from a number of languages, including data from Minnan Chinese, Ju|’hoan, Greek, Japanese, American English, French, Tsimane’, and others (and excluding any data used in the present experiments). Implementation of the voice type classifier is based on the pyannote-audio library (Bredin et al., 2020). Since the diarizer operates directly on the audio waveform, it involves a large amount of computation. However, the voice type classifier supports graphics processing unit (GPU) computing (when available), providing substantial speed-up for the algorithm.

In Lavechin et al. (in press), the voice type classifier was evaluated on a LENA-collected subset of child-centered data also used in the present study. The system achieved diarization F-scores (harmonic mean of precision and recall) of 63.4 for female adult and 42.9 for male adult speech, whereas the corresponding LENA diarizer F-scores were 42.6 and 37.2 for females and males, respectively. In other words, the voice type classifier outperforms the LENA diarization algorithm by a large margin, and can therefore be considered as the state of the art for the present purposes.

### Speech feature extraction

After the audio signal has been segmented into utterances, a number of signal features are extracted from each utterance *i* in order to characterize their structure with a single feature vector **f**_*i*_.

#### Phone recognition

Similarly to LENA (Xu et al., 2008), we explore the use of automatic phone recognition as a feature extractor for ALUC estimation. For this purpose, we use *Allosaurus*^2^, a language-independent phone recognizer (Li et al., 2020). Allosaurus is a multilayer Long Short-Term Memory (LSTM) neural network model trained on 12 different languages for phone and phoneme recognition. Its output consists of a “universal” phone layer with an inventory of phone units that are shared among all training languages, followed by language-dependent mappings from the detected (allo)phones to language-dependent phonemes.

The present system uses an Allosaurus configuration that first produces the most likely string of universal phones and silent breaks for a given input utterance, and then, for dimensionality reduction, maps them onto English phoneme categories that best match with the phone activations. Output phoneme strings are then converted into ternary vowel/consonant/silence categories, and then the total number of vowels, consonants, silent segments, and total of CV and VC alternations are extracted as four numerical features *f*_*i*__,1_ … *f*_*i*__,4_ describing the linguistic characteristics of each input utterance *i*.

#### Syllable count estimation

In addition to using language-independent phone recognition features, we also investigated the use of language-independent syllabification of speech. For this purpose, we utilize the SylNet algorithm (Seshadri & Räsänen, 2019)—an end-to-end neural network syllable count estimator. In short, SylNet is trained to map an input acoustic signal directly onto the corresponding syllable count using training data with known syllable counts, and without manually specifying any intermediate representations between the inputs and outputs. The model architecture consists of 24-dimensional log-Mel spectrum input, followed by a hierarchy of convolutional layers with increasing temporal receptive field sizes, each extracting increasingly complex nonlinear features from the signal. Each intermediate layer and the deepest convolutional layer feed into a shared integrator module (“PostNet”) with a recurrent layer that is responsible for transforming the inputs from the convolutional layers into a final syllable count estimate (see Seshadri & Räsänen, 2019, for details). The output of SylNet is the estimated number of syllables in each input utterance, and this number is directly used as a feature *fi*_gd_,5 in ALUC estimation.

ALICE uses the SylNet baseline model from the original paper that is trained on approximately 10 hours of hand-annotated Estonian and Korean speech, but further adapting the model to the present daylong child-centered data using the standard adaptation procedure of the algorithm (see Experimental Setup for details). In our initial experiments on child-centered audio, SylNet was also compared to syllabifiers from Wang and Narayanan (2007), Räsänen, Doyle, and Frank (2018a), and Räsänen, Seshadri, and Casillas (2018b). Since SylNet systematically showed superior performance to the tested alternatives, we only report ALICE performance, with SylNet as the chosen syllable-based feature extractor.

#### Signal-level features

Besides measuring phone and syllable counts, utterance duration, total signal energy, and the total number of waveform zero-crossings (ZC) were included as additional low-level signal features *f*_*i*__,6_… *f*_*i*__,8_. Apart from duration, which is a strong correlate with all types of linguistic unit counts, the other two low-level features were expected to potentially provide complementary information regarding the amount of speech in the input. Total energy was measured simply as the squared sum of signal waveform amplitudes, and ZC was measured as the total number of sign changes in the waveform. Initial tests also revealed that all three measures were correlated with the tested ALUCs (*r* = 0.68–0.71 for duration, *r* = 0.42–0.44 for total energy, and *r* = 0.52–0.54 for ZC across the three unit types of interest).

### Mapping of features into ALUCs

The final stage in the pipeline consists of a linear mapping from features **f***i*_gd_ to the corresponding ALUCs, where a separate model is used for each linguistic unit of interest. In principle, phone and syllable counts from the feature extraction modules could be directly used as estimates for phonemes and syllables in the data. However, the use of an intermediate linear mapping allows the system to correct any systematic under- or over-estimation in the feature extractor modules, especially since the extractors were not originally optimized for child-centered data. In addition, linear mapping enables the automatic weighted combination of multiple features, which may still be beneficial, given the highly complex and noisy data.

In ALICE, the linear model for word count estimation is obtained simply as a least squares solution **β**_W_ = **F**^**-1**^**w**, where **w** represents the word counts of the training utterances, **F** is a matrix containing the corresponding feature vectors for each utterance (+ a constant term), and **F**^-1^ denotes a regular Moore-Penrose pseudoinverse of **F**. As a result, any new utterance with features **f**_*i*_ can be mapped into estimated word counts as *wi* = **β**W^T^**f**_*i*_. In a similar manner, separate linear models **β**_P_ for phonemes and **β**_S_ for syllables are obtained from a set of training samples.

## Experiments

The overall goal of the experiments was to 1) investigate the overall performance of the proposed system configurations for measuring infant language exposure, and 2) compare the reliability of the phoneme, syllable, and word count estimation approaches. The experiments were conducted in two basic scenarios: within-corpus generalization and cross-corpus (cross-language) generalization experiments. The former describes how accurately each system variant can perform when adapted to the language or dialect represented by the corpus—a process that requires representative audio data with annotated phoneme/syllable/word counts (cf. WCE-R)—and then tested on a previously unseen participant from the same corpus. The latter scenario describes how well the system performs “out- of-the-box” on any given corpus of any language (cf. LENA). While the corpus-specific setup may provide baseline ALUC performance attainable with the given approach (constrained by the given amount of domain-matching training data), the corpus-independent system represents the user- friendly use case, and hence is also the most likely use scenario of ALICE in practical child language research, particularly in the case of low-resource languages.

In addition, ALICE was evaluated using two conditions for broad class speaker diarization: 1) oracle diarization based on annotated ground truth speaker IDs and utterance boundaries (i.e., manually annotated speech segments), and 2) automatic diarization using the voice type classifier. The former evaluates the accuracy at which different linguistic units can be measured from child- centered recordings using the present feature extractors and the linear mapping approach, assuming perfect speaker diarization. The latter, more realistic condition reflects the expected performance of ALICE when it is being used on raw audio out of the box; in other words, in this latter case, the performance of different ALUCs is necessarily intertwined with the success of the diarization algorithm on the given data.

### Data

The data for our experiments consisted of seven child-centered daylong audio recording corpora. These include the Bergelson corpus (“BER”) from US English-speaking families from New York state (Bergelson, 2016), the LuCiD Language 0–5 corpus (“L05”) consisting of UK English-speaking families from Northwest England (Rowland et al., 2018), the Casillas Tseltal corpus (“TSE”) of Tseltal-speaking families from a rural Mayan community in Southern Mexico (Casillas, Brown, & Levinson, 2017a), the McDivitt and Winnipeg corpora (so-called McDivitt+; here “MCD”) of Canadian English-speaking families (McDivitt & Soderstrom, 2016), the Warlaumont corpus (“WAR”) of US English-speaking families from central California (Warlaumont et al., 2016), the Rosemberg corpus (“ROS”) of Argentinian Spanish-speaking families from the Buenos Aires metropolitan area (Rosemberg et al., 2015), and the Casillas Yélî corpus (“YEL”) of Yélî Dnye- speaking families from a remote island community in Papua New Guinea (Casillas, Brown, & Levinson, 2017a).

The data consist of 4–16-hour at-home recordings from microphones worn by children under age three from diverse linguistic and socioeconomic contexts. BER, MCD, L05, and WAR recordings were collected with the LENA recorder, while the Casillas corpora (TSE and YEL) were recorded with an Olympus WS-382 or WS-852 recorder, and ROS was recorded with a mix of Olympus, Panasonic, Sony, and LENA recorders.

In our experiments, we use the subset of each corpus that has been sampled and manually annotated as a part of the ACLEW project (Bergelson et al., 2017). From each corpus, daylong recordings of 10 target children between the ages of 0 and 36 months with a representative spread of assigned sex and maternal education were chosen for manual annotation. Fifteen 2-minute non- overlapping segments were randomly sampled from each daylong audio for manual annotation (i.e., 300 minutes of audio per corpus), except Tseltal, for which nine 5-minute clips and Yélî Dnye for which nine 2.5-minute clips were randomly extracted. This translates to a total of 36.25 hours of annotated audio, with an approximate average of 10 minutes of annotated speech per target child on all but the TSE corpus, where there is approximately 50% more annotated audio per target child. For MCD, one participant was sampled twice due to an error (but using recordings from two different days). MCD contains small amounts of French, WAR and TSE contain small amounts of Spanish, and YEL contains small amounts of English, all because of bilingual child language environments or linguistic borrowing.

All sampled 2-, 2.5-, and 5-minute segments were manually annotated for utterance boundaries, speaker identity, addressee information (adult vs. child-directed), and vocal maturity of child vocalizations using exactly the same annotation protocol specifically developed for the present type of daylong child-centered data (Casillas et al., 2017b). Each corpus was annotated by a person proficient in the target language, and all annotators were trained to transcribe speech corresponding to what was actually said instead of the canonical lexical forms. In addition, before they could take part in the annotation of actual data, annotators had to prove that they could transcribe at the highest standard, with a high degree of similarity to a representative set of age- and difficulty-variable gold- standard annotation clips.

Gold-standard phoneme and syllable counts were obtained by automatic phonemization and syllabification of the hand-annotated orthographic transcripts. First, the transcripts were cleaned of all non-lexical entries such as incomprehensible speech, non-linguistic communicatives and other non-speech sounds (e.g., &=yawns), and paralinguistic markers (e.g., <hello> [=! sings] to denote singing while speaking). The resulting word strings were then converted into sequences of phonemes either using the Phonemizer tool^3^ (for English) or using hand-crafted letter sequence-to-phoneme conversion rules that, while not reflecting a one-to-one-match between letters and phones, preserved the phonotactic constraints for syllable onsets in each non-English language tested (Spanish, Tseltal, Yélî Dnye). Finally, phoneme strings were syllabified either using the Festival toolkit^4^ tokenizer (for English corpora) or using the maximum onset principle (see, e.g., Kahn, 1976) with language-specific lists of valid syllable onsets, again hand-crafted to work with the phone-converted text (for Spanish, Tseltal, and Yélî Dnye). Gold-standard word counts were derived from the cleaned-up transcripts using white spaces as word boundaries.

Some recordings in BER, and all recordings in TSE, YEL, MCD, and WAR are available from the HomeBank repository (VanDam et al., 2016).

### Evaluation metrics

The performance of the ALUC estimation process was evaluated using two complementary metrics. The first metric is standard linear correlation *r* ∈ [−1, 1] between gold-standard adult phoneme/syllable/word counts *N*_ref,*s*_ and estimated corresponding counts *N*_hypo,*s*_ for each speech segment *s*. The second metric was the median absolute relative error (*ERR*_median_) across the speech segments (Räsänen et al., 2019), defined as

1 $$ {\mathrm{ERR}}_{\mathrm{median}}\left(\%\right)=\mathrm{Median}\left(\frac{\left|{N}_{\mathrm{hypo},s}-{N}_{\mathrm{ref},s}\right|}{N_{\mathrm{ref},s}}\right)\ast 100 $$

Median was used instead of mean, as the absolute relative error distribution can be highly skewed (Räsänen et al., 2019). For both metrics, each segment *s* corresponded to an annotated continuous 2-minute audio clip from the given corpus. In *ERR*_median_ calculation, segments without any annotated syllables or words were ignored to avoid zeros in the denominator.

In short, correlation describes whether the ALUC estimation algorithm is sensitive to *relative* differences in ALUCs across the given pool of test subjects, but does not show whether the *absolute* unit counts are accurate. In contrast, *ERR*_median_ reveals whether the absolute counts are accurate, but is not as descriptive of the relative increases in estimated counts with an increasing amount of true counts across the full range of possible values. Since ALICE and both metrics are deterministic for any given data set, we report the numbers without measures of uncertainty (all reported correlations being *p* < .001).

### Experimental setup

The basic procedure was leave-one-subject-out (LOSO) cross-fold evaluation for within-corpus evaluations, and leave-one-corpus-out (LOCO) for cross-corpus evaluations. In both cases, all but one of the target children’s recordings (LOSO; or corpora for LOCO evaluations) were used to estimate the linear models for the ALUCs of interest using the diarized speech segments from adult talkers and their linguistic unit counts as the training data. Training was done by using the hand- annotated utterance boundaries and speaker IDs, as this was found to lead to slightly higher performance than using diarization-based training segments. The resulting models were then applied to the diarized data on the left-out target child (LOSO; or corpus for LOCO) to get the ALUC estimates for each signal in that fold. The ALUC estimates from all diarized segments corresponding to each 2-minute annotated clip were then summed, and compared to the gold standard according to the metrics described above. The reported results correspond to the average performance across the folds. For overall performance measures comparing different ALICE feature extractor configurations and units to be estimated, the results from the four varieties of English (BER, SOD, WAR, LUC) were first averaged to have one summary performance metric for English (“ENG”). The mean performance across ENG, Argentinian Spanish (“SPA”), Tseltal (“TSE”), and Yélî Dnye (“YEL”) was then calculated. This procedure was utilized in order to avoid biasing the metrics towards English, as four of the seven corpora were different varieties of English. Corpus- specific results are also reported separately using a full set of signal features.

The performance of ALICE was also compared to LENA adult word counts (AWCs) when possible. LENA AWCs were only available for MCD, BER, L05, and WAR corpora (all English), as TSE, ROS, and YEL had been recorded with a mix of other recorders. Note that the 2-minute clips chosen for the manual annotation do not neatly correspond to adult segments detected by LENA, for which LENA calculates corresponding AWCs. Reference LENA AWCs for the clips were derived from the LENA outputs as follows: First, it was assumed that the words detected by LENA were uniformly spaced across the duration of the corresponding adult segment. The words of the segment were then assigned to the 2-minute clip in proportion to their overlap with the clip. For instance, if 50% of a LENA-detected adult segment with 10 LENA-estimated words overlapped with the clip, 5 words were added to the LENA AWC estimate for that clip. Also note that a substantial majority of the LENA segments were fully within the annotated clip limits, as typical adult segments are much shorter than the annotated clips in question, and therefore this approximation affects only 1.3% of all adult segments.

SylNet features were calculated after adapting the baseline SylNet model to the training data of each fold using the gold-standard syllable counts as the reference and following the adaptation procedure described in Seshadri and Räsänen (2019). Allosaurus was used as provided in the original Python library.

### Results

#### Experiment #1: Within-corpus generalization performance

**Fig. 2:**
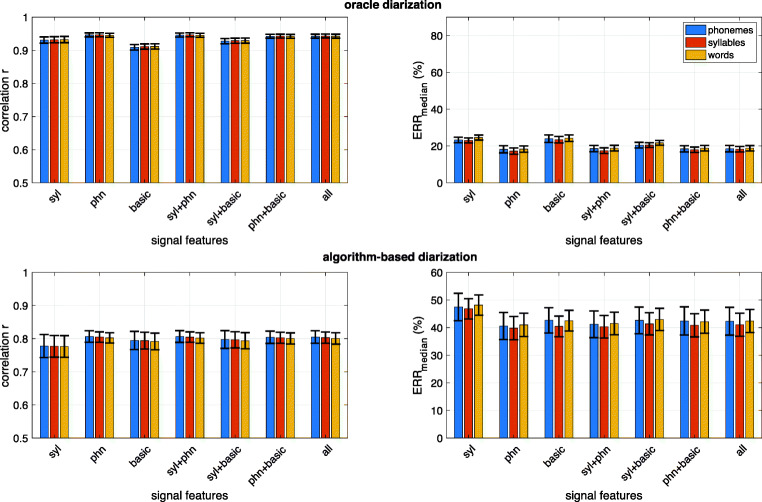
Results from within-corpus experiments for different feature sets and the three types of linguistic units to estimate phonemes, syllables, and words (denoted with different color bars). Results shown are averages across English, Spanish, Tseltal, and Yélî Dnye specific scores. Top panels: oracle diarization based on manual annotation. Bottom panels: actual diarization with an automatic algorithm. Left panels: correlation between gold-standard and estimated linguistic unit counts. *X*-axis denotes the different signal features and their combinations used by ALICE (*phn* = Allosaurus universal phoneme recognition, *syl* = SylNet syllable counting, *basic* = basic signal-level features, *all* = all features together). Error bars denote ±1 standard error across the seven corpora

**Fig. 3:**
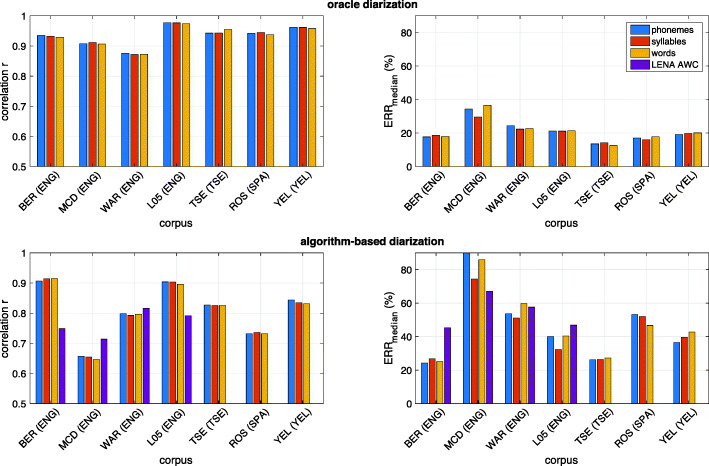
Within-corpus performance of ALICE for different corpora using the full feature set of ALICE. Top panel: oracle diarization. Bottom panel: algorithm-based diarization. LENA adult word count estimation performance is also shown as a reference with violet bars

Figure 2 shows the overall ALICE performance across the three tested languages when training and testing data come from the same corpus (but different recorded participants). The results are shown as a function of signal features used in the estimation process, and separately for the three types of linguistic units, to estimate phonemes, syllables, and words. Performance metrics are shown separately for the case of oracle diarization (top panels) and algorithm-based diarization (bottom panels). Figure 3 shows the performance metrics for each of the seven tested corpora separately and using the full set of tested features (labeled “*all*” in the *X*-axes of Fig. 2), again using the same corpus for training and testing.

#### Discussion of experiment #1

The first experiment tested the ability of ALICE to predict phoneme, syllable, and word counts on a language that ALICE had been previously adapted to (using data from nine subjects). The first finding from the experiment is that all tested feature combinations perform at a similar level. When exact utterance boundaries and talker identities are available (Fig. 2 top panels), correlations between estimated and true ALUCs are highly similar between different feature configurations. While basic signal features are slightly below SylNet in performance, and with Allosaurus features being the best ones, the differences are generally minor. In addition, there are no meaningful differences in the accuracy at which different linguistic units can be estimated. Relative error (*ERR*_median_) measures follow the same trend. When comparing the different corpora (Fig. 3, top panels), there are some differences in terms of MCD and WAR being more challenging than the others in terms of correlation (for WAR) and *ERR*_median_ (for MCD). While the exact reason for this is unclear, they are both corpora where some of the speech is in a different language (French for MCD and Spanish for WAR) from the majority language (English), which may impact the performance. However, TSE and YEL also contain some second language speech (Spanish and English, respectively) but show no noticeable disadvantage in performance. Overall, the experiment shows that if utterances can be accurately extracted and a language-specific ALUC predictor can be trained, all three investigated linguistic unit types are equally measurable.

As expected, when ideal speaker diarization is replaced with an actual diarization algorithm, the accuracy of ALICE decreases across the board (Figs. 2 and 3 bottom panels). While the feature sets are still performing at a comparable level, corpus-specific differences are more prominent. This is likely due to varying diarization performance in different recording environments of different corpora. The average correlation between estimated and true units is *r* = 0.80 and *ERR*_median_ = 42.31% for phonemes, *r* = 0.80 and *ERR*_median_ = 41.05% for syllables, and *r* = 0.80 and *ERR*_median_ = 42.39% for words when using the full feature set. Comparing these figures to LENA AWC performance (Fig. 3, bottom panels), ALICE has lower or comparable *ERR*_median_ on three of the four English corpora with LENA baselines. ALICE’s correlation is also substantially higher on BER and L05 but is lower on MCD and WAR. Again, there are no observable differences in performance for measuring different linguistic units. Overall, the results highlight the fact that inaccuracies in speaker diarization have a huge impact on system performance. In addition, when data from only nine subjects are used in the LOSO adaptation procedure for each language, we can speculate that the overall performance may be sensitive to outlier subjects, for which the diarizer may perform very differently compared to the overall population (see also Räsänen et al., 2019, for analyses with voice activity detection).

#### Experiment #2: Cross-corpus generalization performance

**Fig. 4:**
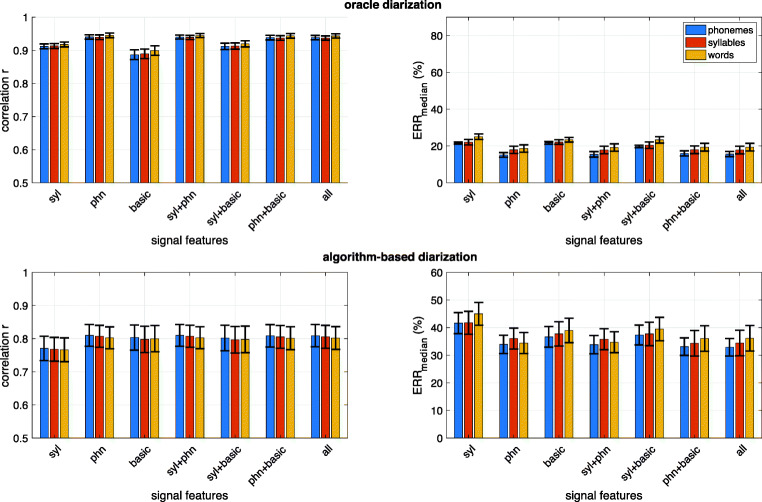
Results from cross-corpus experiments for the different feature sets and the three types of linguistic units to estimate: phonemes, syllables, and words (denoted with different colored bars). Results shown are averages across English, Spanish, Tseltal, and Yélî Dnye specific scores. Top panels: oracle diarization based on manual annotation. Bottom panels: actual diarization with an automatic algorithm. Left panels: correlation between gold-standard and estimated linguistic unit counts. *X*-axis denotes the different signal features and their combinations used by ALICE (*phn* = Allosaurus universal phoneme recognition, *syl* = SylNet syllable counting, *basic* = basic signal-level features, *all* = all features together). Error bars denote ±1 standard error across the seven corpora

**Fig. 5:**
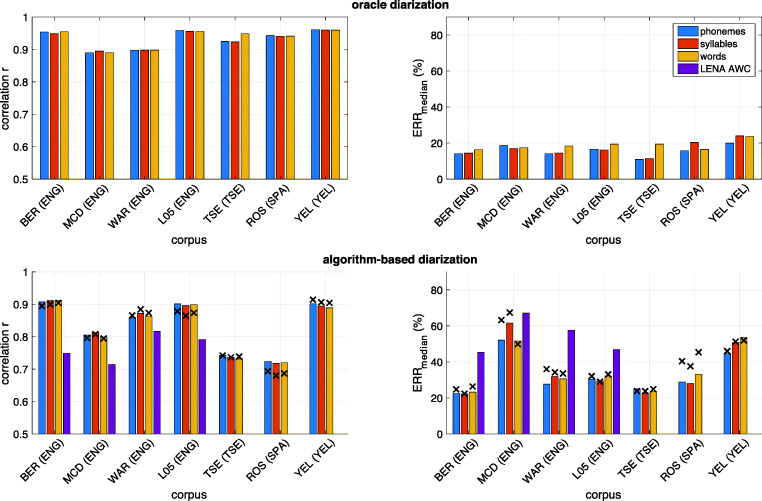
Cross-corpus performance of ALICE for different corpora using the full feature set of ALICE (bars) and using the SylNet+basic features combination used in the final open-source version of the system (black crosses). Top panel: oracle diarization. Bottom panel: algorithm-based diarization. LENA adult word count estimation performance is also shown as a reference with violet bars

While experiment #1 was focused on models adapted to a specific corpus, experiment #2 tested how well ALICE generalizes to new corpora when trained on a number of different corpora consisting of different languages. Figure 4 shows the corresponding results for different feature sets when measured across all four languages, and Fig. 5 shows corpus-specific performance measures when using the full set of ALICE features. In addition, Fig. 5 illustrates system performance when using SylNet and basic signal-level features, as this combination is used in the publicly distributed version of ALICE (see Concluding Remarksfor motivation). Again, top panels of both figures indicate performance with oracle diarization while bottom panels show the performance with the actual diarization algorithm. Finally, Table 1 summarizes the overall cross-language performance (from Fig. 4) for easier comparison.

**Table 1:** Language-independent ALUC estimation performance of ALICE across English, Tseltal, Argentinian Spanish, and Yélî Dnye using different feature sets (rows) and for different linguistic units as targets of estimation (columns). Correlation (*r*) and median relative error rate (*ERR*_median_) metrics are shown for each of the combinations

	Phonemes		Syllables		Words	
	*r*	*ERR* median *%*	*r*	*ERR* median *%*	*r*	*ERR* median *%*
SylNet	0.77	41.63	0.77	41.76	0.77	44.98
Allosaurus	**0.81**	33.91	**0.81**	36.03	**0.80**	**34.41**
Basic	0.80	36.66	0.80	37.72	**0.80**	38.98
SylNet+Allosaurus	**0.81**	33.83	**0.81**	35.81	**0.80**	34.72
SylNet+Basic	0.80	37.31	0.80	37.72	**0.80**	39.49
Allosaurus+Basic	**0.81**	33.11	**0.81**	**34.34**	**0.80**	36.06
All together	**0.81**	**32.90**	**0.81**	34.42	**0.80**	36.15

#### Discussion of experiment #2

The first finding (shown in Fig. 4) is that there is a consistent pattern in the accuracy at which different linguistic units can be estimated cross-linguistically. The error rates are generally lowest for phonemes, followed by syllables, with words having the highest errors. Looking at corpus-specific performance with real diarization (Fig. 5, bottom panels), one can see that the advantage for phonemes and syllables comes primarily from the non-English corpora, where phoneme and syllable estimation in terms of *ERR*_median_ is more accurate for Spanish and Yélî Dnye than the corresponding word estimation accuracies. For English, the results are more mixed, but there is generally a small advantage for phonemes and words over syllables, especially on MCD and WAR. Since at least half of the training data was always English, this demonstrates that ALICE generalization towards novel languages is slightly better for phonemes and syllables, likely since their measurable surface characteristics, including duration, are more universal than those of words (see the second section). However, the overall average differences in different linguistic units are very small.

The second main finding from the second experiment is that there is no significant performance drop from the corpus-specific models of the first experiment. In fact, correlation-based performance on all English corpora (BER, MCD, WAR, L05) and on YEL improves compared to the within-corpus training, and average *ERR*_median_ also improves on all but YEL. The average correlation between estimated and true units is *r* = 0.80 and *ERR*_median_ = 32.90% for phonemes, *r* = 0.80 and *ERR*_median_ = 34.42% for syllables, and *r* = 0.80 and *ERR*_median_ = 36.15% for words when using the full feature set with the diarization algorithm. This implies that whatever is lost in performance due to lack of language-specific information is compensated by the larger pool of subjects available to linear model estimation (60 multi-language vs. 9 language-specific recordings). Comparison to LENA on English corpora (Fig. 5) also shows how ALICE outperforms LENA in all cases and on both metrics. As for the features used (Fig. 4 and Table 1), there are again no major differences in the performance of different feature sets, except that SylNet performs worse than the others when used in isolation. In terms of correlation, even the basic signal-level features (duration, total energy, total zero-crossings) are competitive with the more sophisticated phone recognition and syllable counting models. In terms of relative errors with algorithmic diarization, the best individual feature extractor is the Allosaurus phone recognizer. However, as a drawback, Allosaurus is also the most computationally expensive of all three feature extractors.

In terms of different languages, there is some variation in performance, with English corpora and YEL being generally more accurate to estimate (in terms of correlation). Argentinian Spanish is the most difficult in terms of relative counts, whereas MCD and YEL are the most challenging in terms of *ERR*_median_. The high correlation but poor *ERR*_median_ on YEL also illustrates how the two metrics are complementary to each other. Previous LENA studies have reported correlations between gold-standard and LENA AWCs, such as *r* = 0.64 for French (10-minute segments), *r* = 0.67 for Swedish (5-minute segments), *r* = 0.99 for Finnish (1-hour segments), *r* = 0.88 for Dutch (5-minute segments), *r* = 0.80 for Mexican Spanish (1-hour segments), and *r* = 0.73 for Mandarin Chinese (15-minute segments). The corresponding *ERR*_median_ are 36.5% for French, 59.5% for Swedish, 75.2% for Finnish, 42.9% for Dutch, and 50.2% for Mexican Spanish (the Mandarin Chinese *ERR*_median_ is not available; see Räsänen et al., 2019, for an overview). Since there is a general trend for higher correlations with longer measurement windows (Räsänen et al., 2019), the present results with 2- minute windows (5-minute for Tseltal and 2.5-minute for Yélî Dnye) can be considered as at least comparable to those of LENA. In fact, when all 2-minute clips of each subject are pooled together for an overall ALUC estimate per subject (total 30 minutes of speech; 45 minutes for Tseltal; 22.5 minutes for Yélî Dnye), ALICE output correlations across the four languages range from *r* = 0.81 (ROS) to *r* = 0.95 (WAR) with a mean of *r* = 0.90 for phoneme counts, from *r* = 0.80 (ROS) to *r* = 0.96 (MCD and WAR) with a mean of *r* = 0.90 for syllables, and from *r* = 0.81 (ROS) to *r* = 0.96 (MCD and WAR) with a mean of *r* = 0.91 for words.

As for error analysis, the difference between oracle and algorithm-based diarization (Fig. 5 top and bottom rows) reveals that corpus-specific differences in ALUC estimation accuracy are again primarily driven by differences in diarization performance on these corpora. In the case of ideal diarization, all corpora would have *r* > 0.89 and *ERR*_median_ < 23% for all three units of interest. In reality, the diarization performance may depend on corpus- and infant-level factors such as the distribution of acoustic environments (busy indoor spaces, noisy cars, quiet rural landscapes, etc.) and their influence on the amount and type of electronic speech present in the recordings, the average proportion of overlapping speech in the surroundings, and more. Despite the 200 hours of training data used for the current diarizer, it is still impossible to create a system that generalizes perfectly to all kinds of acoustic environments potentially encountered in naturalistic recordings across variable cultural groups, economies, social environments, and lifestyles (see also Lavechin et al., in press).

Overall, the results show that ALICE is competitive with LENA directly out-of-the-box, with comparable performance to previous literature reports, and clearly outperforms LENA in all cases where direct comparison on the same data was possible in our experiments.

## Conclusions and limitations

This work started by asking what type of linguistic units are the most meaningful measures of child language input, especially when developmental, linguistic, and technical considerations are taken into account. We discussed a number of potential cross-linguistic advantages and disadvantages in measuring phonemes or syllables over words. We also presented a new open-source algorithm for linguistic unit count estimation called ALICE, and then used it to test automatic estimation of all three candidate units with data from seven different corpora of child-centered daylong audio recordings.

The main finding from the experiments was that language-independent phoneme count estimation is somewhat more accurate than estimation of syllable or word counts, and that syllables are also somewhat more accurate to estimate than words. The advantage for smaller linguistic units is greater for languages that differ substantially from the training data, such as generalizing from English-dominated training data to Yélî Dnye or Argentinian Spanish. As shown in the second section, phonemes and syllables are better grounded to the underlying acoustic input by having certain shared characteristics across all languages, and these are what Allosaurus and SylNet attempt to directly measure in a language-independent manner. In contrast, direct measurement of words is not possible without knowing the lexicon of the language: a single language-independent mapping from signal features to word counts is agnostic to whether the input consists of mostly monosyllabic and relatively short-duration words (English) or multisyllabic longer words. The disadvantage of words does not disappear even if we had a representative set of training data from all of the world’s languages, as the resulting model would still tend towards cross-linguistic averages instead of the specific characteristics of any given language. However, the overall differences in the accuracy of the different units with respect to ground truth are small, and hence selection of one unit over the others could also be done based on developmental or experimental considerations . This flexibility offered by ALICE is critical, particularly considering that the type of linguistic units that most robustly predict language development outcomes cross-linguistically are still yet to be substantiated. Similarly, unit flexibility benefits future work correlating unit types with input “quality” measures across grammatically diverse languages, e.g., number of estimated syllables vs. words per utterance as an index of syntactic complexity. Finally, ALICE’s word count estimation was shown to be very competitive with LENA’s—this in addition to its ability to estimate syllable and phoneme counts contributes to the attractiveness of ALICE as an open-source, flexible alternative for automatically measuring the amount of speech in child-centered audio recordings.

### Limitations

Syllable and phoneme counts in the present study were derived from orthographic transcripts using automated pipelines. While we manually specified phonemization and syllabification rules, thereby providing relatively systematic phoneme and syllable counts for the given transcripts, the resulting phonology-based syllable counts should not be taken as a purely error-free gold standard of the syllabic structure of what was actually said (phonetic syllables). Information regarding the detailed style of speech (rhythm, timing, and pronunciation) is lost in the speech-to-transcript and transcript- to-phonemes conversions of the present pipeline, even if the annotators were asked to transcribe what was actually said instead of the corresponding canonical lexical forms. This adds some noise to the gold-standard phoneme and syllable counts, which impacts some of the detailed findings on how accurately they can be measured. However, manual phonetic annotation of the present data from the seven different corpora was beyond the scope of the present efforts, and would require coordinated and sustained effort from a large team of trained annotators.

Another limitation of the study is that, while seven different child-centered corpora were utilized, the data were still strongly biased towards English and contained only three other languages: Argentinian Spanish, Tseltal, and Yélî Dnye. Comparison to a larger set of languages with different characteristics would have provided more information on the cross-linguistic applicability of ALICE and on measurement of different linguistic units. However, our four languages are still relatively distinct: UK/US English, an analytic language, has a large proportion of monosyllabic words (Greenberg, 1999), while Argentinian Spanish, Tseltal, and Yélî Dnye more often use multisyllabic and/or multi-morphemic words (the first two are synthetic: the former fusional and the latter mildly polysynthetic; Polian, 2013; the third’s clitic system and frequent suppletion defies traditional categorization, but 60% of words have two or more syllables; Levinson, under review). The primary reason for using the current set of corpora was that they had all been annotated using exactly the same annotation protocol (Casillas et al., 2017b), ensuring maximal mutual compatibility and comparability in our experiments. We also took some steps to mitigate the impact of English-heavy distribution in our experiments by measuring the overall ALICE performance with an equal weight on the four unique languages. In addition, we experimented with frequency-balanced training of ALICE to see whether that would impact the cross-language generalization performance of the system, but it did not lead to any performance gains over the baseline approach (not reported separately).

Finally, direct performance comparison to LENA was not possible in three of the four tested languages. This was because LENA analysis software outputs were only available for the English corpora, as Tseltal, Argentinian Spanish, and Yélî Dnye data were collected with non-LENA audio recorders; LENA software only accepts audio files that are recorded with the LENA recorder. However, this limitation also underscores the need for free open-source software that can be used for the automatic analysis of linguistic units in child-centered recordings.

### Concluding remarks

The current work focused on theoretical considerations and practical feasibility of measuring phonemes, syllables, and words from child-centered daylong recordings as automatic measures of child language input. However, we did not test the developmental predictive power of these units, but such experiments could be conducted in the future work using ALICE on recordings of child language input together with measurements of language development outcomes; given the cross- linguistic suitability of ALICE, this kind of study with diverse comparative samples is now more feasible.

The shared open-source distribution of ALICE is configured to use SylNet and basic features as the feature extractors. This choice was ultimately motivated by SylNet’s substantially lower computational demands compared to Allosaurus, especially on platforms without support for GPU- based computing, even though this comes at a slight performance cost on some of the tested corpora (see Fig. 5 in Results). The linear models used for ALUC estimation will also be the average models across the four languages in the present study, but can also be customized for language-specific mappings if needed, as documented with the tool.

## Footnotes

1 https://github.com/orasanen/ALICE

2 Allosaurus is available for Python as a pip package called ipa-recognizer. This paper uses version 0.0.3 of the system.

3 https://github.com/bootphon/phonemizer

4 http://www.cstr.ed.ac.uk/projects/festival/
